# Tunicate Heparan Sulfate Enriched in 2-Sulfated β-Glucuronic Acid: Structure, Anticoagulant Activity, and Inhibitory Effect on the Binding of Human Colon Adenocarcinoma Cells to Immobilized P-Selectin

**DOI:** 10.3390/md17060351

**Published:** 2019-06-12

**Authors:** Wallace S. Abreu, Paulo A. G. Soares, Juliana M. Motta, Eliene O. Kozlowski, Felipe C. O. B. Teixeira, Mariana A. Soares, Lubor Borsig, Paulo A. S. Mourão, Mauro S. G. Pavão

**Affiliations:** 1 Programa de Glicobiologia, Instituto de Bioquímica Médica Leopoldo de Meis and Hospital Universitário Clementino Fraga Filho, Universidade Federal do Rio de Janeiro, 21941-913 Rio de Janeiro, RJ, Brazil; wallace.s.abreu@gmail.com (W.S.A.); paulogsoares@hucff.ufrj.br (P.A.G.S.); jmotta@bioqmed.ufrj.br (J.M.M.);felipebrito7@gmail.com (F.C.O.B.T.); mariana.alsoares@gmail.com (M.A.S.); 2 Institute of Physiology, University of Zurich and Zurich Center for Integrative Human Physiology, 8057 Zurich, Switzerland; lborsig@access.uzh.ch

**Keywords:** heparan sulfate, cancer, coagulation, ascidian glycosaminoglycans, P-selectin, marine biology, nuclear magnetic resonance

## Abstract

Heparin or highly sulfated heparan sulfate (HS) has been described in different invertebrates. In ascidians (Chordata-Tunicata), these glycosaminoglycans occur in intracellular granules of oocyte accessory cells and circulating basophil-like cells, resembling mammalian mast cells and basophils, respectively. HS is also a component of the basement membrane of different ascidian organs. We have analyzed an HS isolated from the internal organs of the ascidian *Phallusia nigra*, using solution ^1^H/^13^C NMR spectroscopy, which allowed us to identify and quantify the monosaccharides found in this glycosaminoglycan. A variety of α-glucosamine units with distinct degrees of sulfation and *N*-acetylation were revealed. The hexuronic acid units occur both as α-iduronic acid and β-glucuronic acid, with variable sulfation at the 2-position. A peculiar structural aspect of the tunicate HS is the high content of 2-sulfated β-glucuronic acid, which accounts for one-third of the total hexuronic acid units. Another distinct aspect of this HS is the occurrence of high content of *N*-acetylated α-glucosamine units bearing a sulfate group at position 6. The unique ascidian HS is a potent inhibitor of the binding of human colon adenocarcinoma cells to immobilized P-selectin, being 11-fold more potent than mammalian heparin, but almost ineffective as an anticoagulant. Thus, the components of the HS structure required to inhibit coagulation and binding of tumor cells to P-selectin are distinct. Our results also suggest that the regulation of the pathway involved in the biosynthesis of glycosaminoglycans suffered variations during the evolution of chordates.

## 1. Introduction

Heparan sulfate (HS) is a sulfated glycosaminoglycan present in virtually all animal species [1,2]. It has a complex and diverse structure, which varies according to cell type and differentiation. Mammalian HS chains are formed by repeating disaccharide units, consisting of β-D-glucuronic acid (GlcA) or α-L-iduronic acid (IdoA) 1→4-glycosidically linked to α-D-glucosamine (GlcN). This disaccharide is usually highly modified. The hexuronic acid residues can be 2-sulfated, mostly in IdoA and rarely in GlcA. The GlcN residue can be *N*-acetylated or *N*-sulfated (GlcNAc or GlcNS), and may also contain 6-O-sulfo and/or 3-O-sulfo groups. Heparin, on the other hand, is less heterogeneous made up of repeating disaccharide units of hexuronic acid (IdoA or GlcA) linked 1→4 to GlcN units. The majority of the uronic acid residues occur as IdoA containing a 2-O-sulfo group, and the GlcN is mostly *N*-sulfated and 6-sulfated. Small contents of GlcN may contain a 3-O-sulfo group [3,4].

In ascidians (Chordata-Urochordata), also known as sea-squirts, solitary species like the stolidobranchia *Styela plicata* and the phlebobranchia *Phallusia nigra* are rich in glycosaminoglycans such as dermatan sulfate (DS) and heparin/HS [5,6,7,8]. In both ascidians, the DSs are formed by disulfated disaccharide units composed exclusively by IdoA 2-sulfated. On the other hand, the *N*-acetyl β-D-galactosamine (GalNAc) residues are sulfated at position 4 or 6, depending on the species. In *S. plicata* the GalNAc are mostly 4-sulfated whereas in *P. nigra*, GalNAc 6-sulfated units prevail [6,7].

The structure of the heparin/HS polymers isolated from *S. plicata* had been previously studied by disaccharide analysis and multi-dimensional nuclear magnetic resonance (NMR) techniques. These studies revealed that the disaccharide units of the ascidian heparin/HS are formed exclusively by IdoA 2-sulfated, linked to *N*-sulfated GlcN with different percentages of 6-sulfation (38–100%). Small percentage of the *N*-sulfated GlcN can be 3-sulfated or 3- and 6-disulfated (3.8–9.8%) [9].

In this paper, we employed different nuclear magnetic resonance (NMR) spectroscopy techniques to characterize the heterogeneous structure of the HS isolated from the phlebobranchia ascidian *P. nigra* and estimated the ability of the glycan to inhibit coagulation and the binding of human colon adenocarcinoma cells to immobilized P-selectin. We showed that the ascidian HS is particularly enriched in 2-sulfated GlcA and 6-sulfated *N*-acetylated GlcN units; it does not inhibit coagulation but is 11-fold more active than mammalian heparin as an inhibitor of the binding of human colon adenocarcinoma cells to P-selectin.

## 2. Results and Discussion

### 2.1. Extraction and Purification of the P. nigra Heparan Sulfate

**Figure 1:**
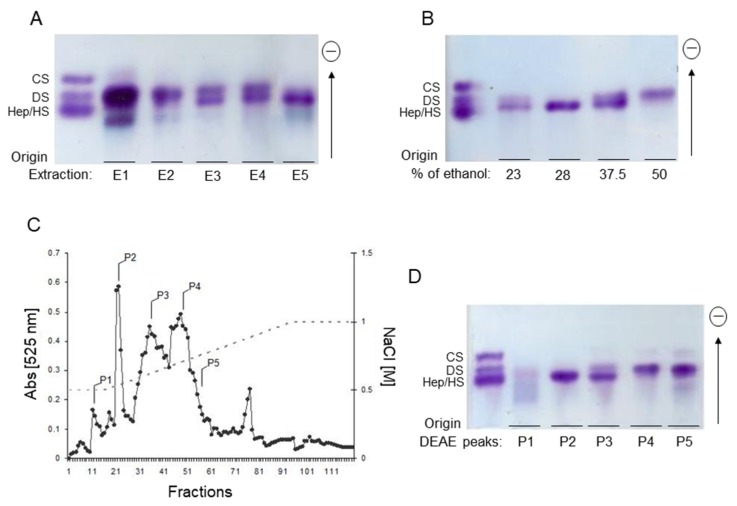
Fractionation and analysis of glycosaminoglycans from the viscera of *P. nigra*. (**A**) Agarose gel electrophoresis of the glycosaminoglycans extracted from the ascidian viscera by five successive proteolytic digestions (E1→E5). (**B**) Glycosaminoglycans from the third and fourth extractions (E3 + E4) were pooled, precipitated by ethanol using the concentrations indicated in the panel and analyzed by agarose gel electrophoresis. (**C**) Glycosaminoglycans from the second and third extractions (E2 + E3) were pooled (~20 mg) and applied to a DEAE-cellulose column. The column was developed by an increasing concentration of NaCl (0.5→1.0 M), and the fractions analyzed by metachromasia, as described under Material and Methods. Fractions under the peaks (P1→P5) were pooled as indicated in the figure, dialyzed against distilled water, lyophilized, and analyzed by agarose gel electrophoresis (**D**). Fraction P2 contains the purified HS from *P. nigra*.

Total glycans from *P. nigra* were extracted from the viscera (internal body) of the ascidian *P. nigra* by five successive proteolytic digestions. Glycans obtained in each one of extractions were recovered separately by cetylpyridinum chloride/ethanol precipitation and analyzed by agarose gel electrophoresis, as shown in Figure 1A. In the first extraction (E1), three metachromatic bands were observed. A low-intensity band with high mobility, migrating as standard chondroitin sulfate (CS); a major metachromatic band with intermediate mobility, migrating as standard DS; and finally, a low-mobility band with no correlation with any standard glycosaminoglycans used, which is hardly observed in the subsequent extractions (E2–E5). E2, contains mostly the metachromatic band with the same mobility as standard DS. E3 and E4 contain two main metachromatic bands, a high-mobility band, migrating as DS and a low-mobility band, migrating between standard DS and HS, which is the sole material observed in E5.

The metachromatic band migrating as standard DS, observed in E1 (highly intense staining), E2, E3, and E4, and absent in E5, corresponds to an oversulfated DS, previously characterized, formed mostly by repeating disaccharide units composed by 2-sulfated α-IdoA and 6-sulfated β-GalNAc [6]. To purify the glycan corresponding to the low-mobility metachromatic band in E3 and E4, which migrates between standard DS and HS, the material from E3 and E4 were pooled, lyophilized, and suspended in a 2% NaCl solution. The glycans were then precipitated selectively by increasing NaCl concentrations and analyzed by agarose gel electrophoresis (Figure 1B). The low-mobility metachromatic band, migrating between standard DS and HS, was precipitated free of contaminants with ethanol 28%. Similarly, the oversulfated DS, previously characterized, was precipitated free of contaminants with 50% ethanol. On the other hand, the precipitates obtained with ethanol at 23% and 37.5% contain both low- and high-mobility metachromatic bands.

Alternatively, a mixture of E2 and E3 was fractionated by ion-exchange chromatography on a diethylaminoethyl (DEAE)-cellulose column, eluted with a linear gradient of 0.5→1.0 M NaCl, as described under Material and Methods (Figure 1C). Several metachromatic peaks were eluted from the column. The fractions under peaks denominated as P1 to P5 were pooled and analyzed by agarose gel electrophoresis (Figure 1D). The high-mobility metachromatic band corresponding to the previously characterized oversulfated DS [6] was eluted from the column at high NaCl concentrations, 0.75 M and 0.8 M NaCl (peaks P4 and P5, respectively). The low-mobility band migrating between standard DS and HS was eluted from the column in a homogeneous form at 0.6 M NaCl (P2 in Figure 1C).

**Figure 2:**
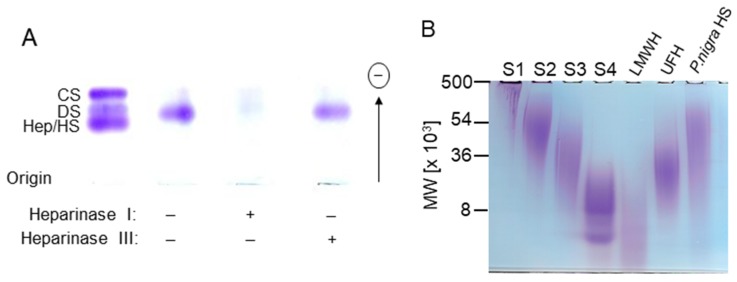
Digestion of the heparan sulfate by heparin lyases and determination of the molecular weight by polyacrylamide gel electrophoresis. (**A**) The purified HS from *P. nigra* (~20 µg) was incubated with heparinase I or III and, at the end of the incubation period, mixtures were analyzed by agarose gel electrophoresis. (**B**) *P. nigra* HS and standards of known molecular mass were applied to a 6% polyacrylamide slab gel and after electrophoresis stained with toluidine blue. The molecular mass markers used were: dextran sulfate 500 (S1; average molecular weight, ~500 kDa), CS from shark cartilage (S2; ~54 kDa); CS from whale cartilage (S3; ~36 kDa); dextran 8 (S4; ~8 kDa); low molecular weight heparin (LMWH) (~8 kDa) and unfractionated heparin (UFH) (~17 kDa).

Agarose gel electrophoresis, before or after incubation with specific heparin lyases (Figure 2A), revealed that the metachromatic material obtained in P2 is a heparin/HS-like glycan. Thus, P2 resists degradation with heparinase III, but is completely degraded after incubation with heparinase I. Heparinase I cleaves hexosaminidic linkages between hexuronic 2-sulfated and glucosamine *N*-sulfated, whereas heparinase III cleaves preferentially hexosaminidic linkages between *N*-acetylated glucosamine and non-sulfated glucuronic acid [10]. Our results suggest that the disaccharide units in the *P. nigra* HS may contain a high content of *N*-sulfated GlcN and 2-sulfated hexuronic acid residues. This was further evaluated by analysis of the monosaccharides found in the ascidian HS using NMR spectroscopy.

Polyacrylamide gel electrophoresis in sodium barbital was used to estimate the molecular weight of the *P. nigra* HS (fraction P-2) (Figure 2B). Based on comparison with electrophoretic motilities of molecular mass markers, the ascidian HS has a very polydisperse mass with an average of molecular weight of ~50 kDa.

### 2.2. Structural Characterization of the P. nigra Heparan Sulfate by NMR

**Figure 3:**
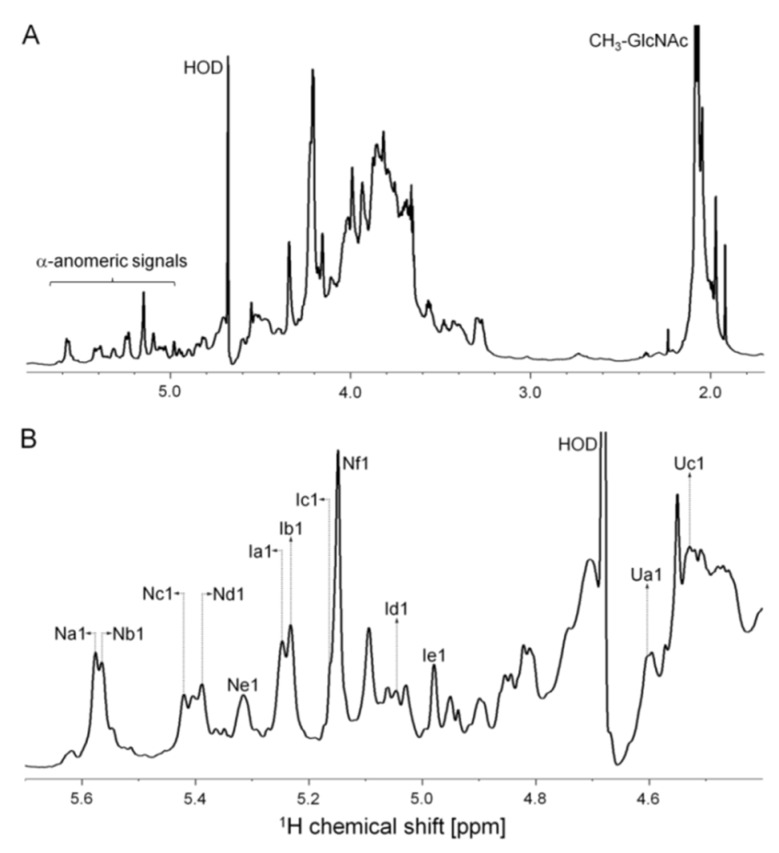
1D ^1^H nuclear magnetic resonance (NMR) spectrum at 900 MHz of *P. nigra* heparan sulfate. (**A**) Expansion of the 5.8–1.8 ppm region of the spectrum and (**B**) enhancement of the anomeric signals region. Labels in (B) indicate signals of the α-anomeric protons of *N*,6-disulfated (Na and Nc), *N*-sulfated (Nb and Ne), *N*-acetylated (Nd), and 6-sulfated *N*-acetylated (Nf) α-GlcN units; 2-sulfated (Ia, Ib, and Ic) and non-sulfated (Id and Ie) α-IdoA units; 2-sulfated (Ua and Ub) and non-sulfated (Uc and Ud) β-GlcA; and CH_3_ of the *N*-acetyl group from α-GlcN.

**Figure 4:**
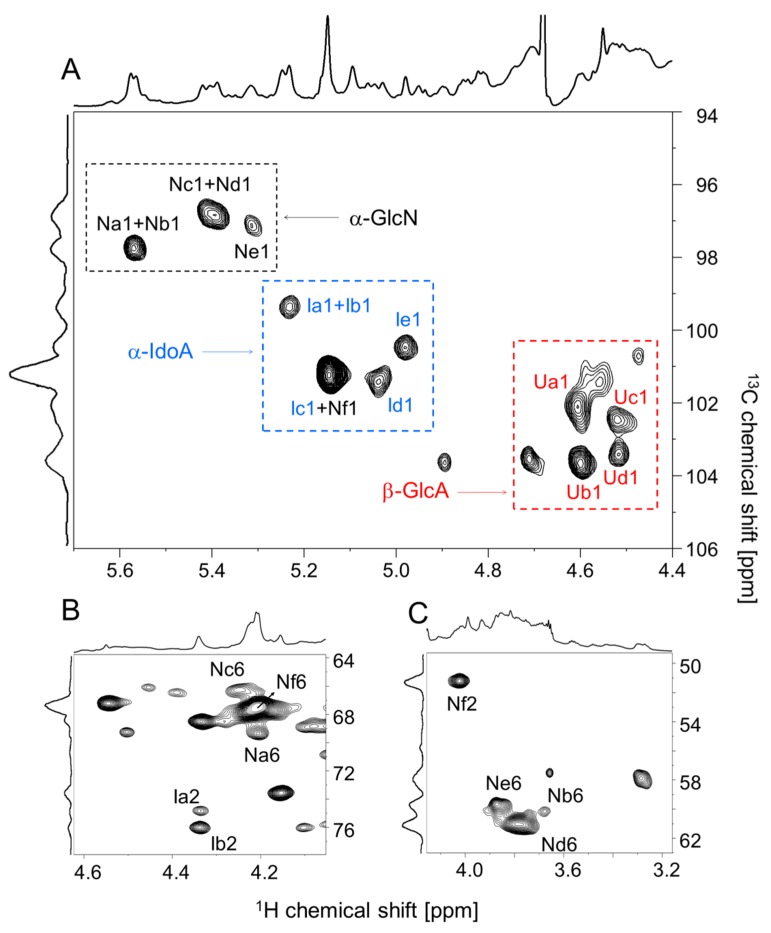
^1^H/^13^C Ed-HSQC spectra at 900 MHz of *P. nigra* heparan sulfate. (**A**) Strip of the anomeric and (**B**,**C**) of the H2/C2 e H6/C6 regions of the spectrum. Labels in (A) indicate signals of the anomeric protons of *N*,6-disulfated (Na and Nc), *N*-sulfated (Nb and Ne), *N*-acetylated (Nd), and 6-sulfated *N*-acetylated (Nf) α-GlcN units; 2-sulfated (Ia, Ib, and Ic) and non-sulfated (Id and Ie) α-IdoA units; 2-sulfated (Ua and Ub) and non-sulfated (Uc and Ud) β-GlcA units; and CH_3_ of the *N*-acetyl group (CH_3_ from N-acetylated GlcN). Dotted squares in the panel (A) enclose the regions of the spectra containing the anomeric signals of α-glucosamine (α-GlcN, in black), α-iduronic acid (α-IdoA, in blue), and β-glucuronic acid (β-GlcA, in red).

**Figure 5:**
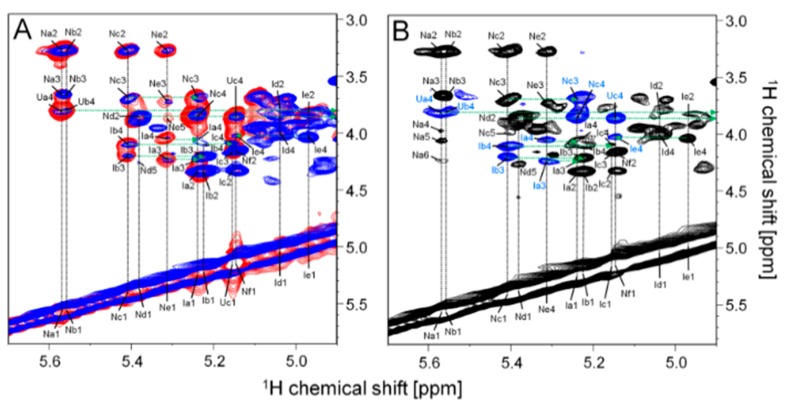
2D ^1^H-^1^H TOCSY/NOESY and phase-sensitive TOCSY spectra of *P. nigra* heparan sulfate. (**A**) Strips from the ^1^H-^1^H TOCSY (in blue) and NOESY (in red) spectra of the anomeric region of the *P. nigra* HS. The intra- and the inter-spin systems are indicated by vertical dashed black lines and horizontal dashed green arrows, respectively. (**B**) ^1^H-^1^H phase-TOCSY spectrum of the HS with in-phase intra-residue spin systems in black (vertical dashed black lines) and anti-phase inter-residue spin systems in blue (horizontal dashed green arrows).

The structure of *P. nigra* HS was further investigated by solution one-dimensional (1D) and two-dimensional (2D) NMR analysis at 900 MHz. 1D ^1^H spectrum reveals a variety of typical anomeric signals of α-GlcN and α-IdoA with chemical shifts similar to values reported in the literature for heparin/HS [9,11,12,13,14] (Figure 3). These signals are clearly visualized on the expansion shown in Figure 3B. The strip of the anomeric region of the 2D ^1^H/^13^C edited-heteronuclear single quantum coherence spectroscopy (Ed-HSQC) (Figure 4A) confirms these assignments. 2D ^1^H-^1^H total correlation spectroscopy (TOCSY), nuclear overhauser effect spectroscopy (NOESY) and phase-TOCSY spectra (Figure 5) completed the assignment of the spin systems of these units. This approach allowed us to determine the sequence and linkage position through the correlation of intra- and inter-nuclear overhauser effects (NOEs) signals in the NOESY spectrum and intra- and inter-rotating-frame overhauser signals (ROEs) in the phase-sensitive TOCSY spectrum [15]. The values of ^1^H and ^13^C chemical shifts of the various units are shown in Table 1.

Six spin systems ascribed to α-GlcN (N-units) were identified and named Na→f. Their chemical shifts are shown in Table 1. Units Na and Nc are *N*,6-disulfated α-GlcN residues and the minor gap (−0.15 ppm) between the chemical shifts of their anomeric protons (5.57 ppm for unit Na and 5.42 ppm for unit Nc) is caused by the distinct neighbor hexuronic acid unit: 2-sulfated α-IdoA in unit Na and 2-sulfated β-GlcA in Nc. This is clearly observed on the ^1^H-^1^H NOESY and phase-TOCSY spectra, which show NOEs and ROEs between H1 of unit Na and H3/H4 of the 2-sulfated β-GlcA residues (unit Ua/b) (see green broken lines in the Figure 5A,B). These connectivities are not observed for residue Nc, which in contrast shows NOEs and ROEs between H1 of the α-GlcN and H3/H4 of the 2-sulfated α-IdoA units. Similar observation is extended to residues Nb and Ne ascribing to *N*-sulfated α-GlcN, linked to either 2-sulfated β-GlcA or 2-sulfated α-IdoA.

The two remaining spin systems of α-GlcN seen in the spectrum of ascidian HS are *N*-acetylated α-GlcN (residues Nd and Nf) linked to non-sulfated β-GlcA. Particularly interesting is the preponderant Nf unit, containing 6-sulfated, *N*-acetylated α-GlcN, which promotes the high field shift of the anomeric proton (~0.23 ppm) compared to the non-sulfated unit (residue Nd). This observation is in line with other data reported in the literature [13,14]. The presence of a high proportion of *N*-acetylated α-GlcN in the ascidian HS is also confirmed by the intense *N*-acetyl signal at 2.06 ppm (Figure 3A).

The identity of the α-IdoA residues were also easily identified especially based on the ^1^H-^1^H spectra (Figure 5). The characteristic ~0.6 ppm downfield shift observed on the sulfation sites indicates that units Ia, Ib, and Ic are 2-sulfated while units Id and Ie are non-sulfated. Furthermore, the NOEs and ROEs observed in the NOESY and phase-TOCSY indicate clearly their neighbor α-GlcN units, as discussed above.

Finally, it remains to assign the β-GlcA units, which are superimposed by the signal of residual water in the ^1^H spectra. In this case we took advantage of the ^1^H-^13^C Ed-HSQC spectrum (Figure 4A) and comparison with literature data [13,14]. Four types of β-GlcA units that differ by the occurrence of 2-sulfation or their neighbor α-GlcN residue were identified, as indicated in Table 1C. Especially relevant is the abundance of 2-sulfated β-GlcA units, which account for ~60% of total β-GlcA residues.

The proportions of the various monosaccharide units found in the ascidian HS were quantified based on the anomeric signals detected on the ^1^H-^13^C Ed-HSQC spectrum (Figure 4A), as reported in Table 1. Some of these signals, such as those of Na1 and Nb1 from the α-GlcN units, were not separated on this region of the spectrum. Quantifications of the superimposed α-GlcN and α-IdoA signals were obtained by integration of their H6/C6 and H2/C2 signals, respectively, subtracted from their correspondent anomeric signal (Figure 4B,C).

Overall, the combination of 1D/2D NMR and enzymatic analysis with heparin lyases suggests that the *P. nigra* HS has a very heterogeneous disaccharide organization, with few heparinase III-sensitive sites randomly distributed throughout the polymer, and without the typical *N*-sulfated domains separated by long *N*-acetyl-rich sequences that are markedly deficient in sulfate groups observed in mammalian HS [3,16].

Several structural features of the *P. nigra* HS deserve attention, for example, the high content of 2-sulfated β-GlcA and 6-sulfated α-GlcNAc residues. During HS biosynthesis in mammals, 2-sulfation of hexuronic acid residues occurs preferentially after α-GlcN *N*-deacetylation/*N*-sulfation and C5-epimerization of GlcA into IdoA. Then, 6-sulfation and, to a lesser extent, 3-sulfation in the α-GlcN takes place [17]. In the ascidian HS, the presence of 2-O-sulfo groups in one-third of the β-GlcA residues (Table 1), indicates that the 2-sulfate sulfotransferase does not necessarily require the action of the C5-epimerase, contrary to what is observed in mammals. Interestingly, although 53% of the β-GlcA is linked to *N*-acetylated α-GlcN, as would be expected in the case of the mammalian pathway, described above, 39% of this α-GlcN contains 6-sulfate groups (Table 1), indicating that the corresponding tunicate 6-sulfotransferase does not require the previous actions of *N*-deacetylase/*N*-sulfotransferase, C5-epimerase, and 2-sulfotransferase. Additionally, the significant quantities of non-sulfated α-IdoA linked to *N*-acetylated, 6-sulfated α-GlcN or to *N*-sulfated α-GlcN (Table 1), detected in the ascidian HS suggests a low substrate specificity of the sulfotransferases involved in the sulfation of hexuronic acid and α-GlcN residues. Finally, the occurrence of small but significant amounts of non-sulfated α-IdoA residues linked to *N*-acetylated α-GlcN (Table 1) suggests a striking feature of the invertebrate HS C5-epimerase in acting upon non-substituted α-GlcN residues.

Furthermore, these results suggest that the enzymes involved in chain modification during HS biosynthesis in *P. nigra* may have a different sequential organization in the Golgi or differ in substrate specificity from their mammalian counterparts. Although there is not much information about the enzymes involved in the biosynthesis of HS in ascidians, the results of the present work are in line with other reports showing that tunicates contain a variety of unique polysaccharides, including glycosaminoglycans with distinct sulfation patterns compared to their mammalian counterparts [6,7,9]. In fact, *P. nigra* contains an oversulfated DS composed mainly by disaccharide units of 2-sulfated α-IdoA and 6-sulfated β-GalNAc [6]. This glycan has no anticoagulant activity but presents a significant anti-P-selectin activity, which is responsible for a drastic attenuation of experimental metastasis in animals. Interestingly, this compound can be easily isolated from the ascidian tissues as reported here (Figure 1A,B) and in previous work of our group [6]. Fractions enriched in both 2,6-disulfated DS and HS can be isolated free of contaminants by a simple enzymatic extraction and ethanol precipitation (fractions E3 and E4, Figure 1A), providing interesting material for further studies to investigate, for instance, the anti-metastatic potency of the glycans.

### 2.3. Heparan Sulfate from P. nigra Has Almost No Effect on Coagulation

**Figure 6:**
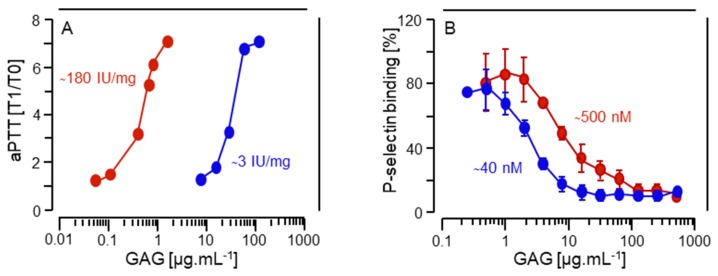
In vitro anticoagulant activity and P-selectin binding inhibition by *P. nigra* heparan sulfate. (**A**) Anticoagulant activity of *P. nigra* HS and standard heparin (blue and red circles, respectively, in both panels) assessed by aPTT assays performed in human plasma with increasing concentrations of the glycosaminoglycans. Results are expressed as ratios of clotting time in presence (T1) and absence (T0) of the glycosaminoglycans fitted as second-order polynomial curves. Anticoagulant potencies as IU·mg^−1^ were calculated based on curves fitted to the 6th International Heparin Standard. (**B**) Inhibitory effect of *P. nigra* HS or heparin on adhesion of LS 180 cells onto immobilized P-selectin. P-selectin was immobilized on a 96-well microplates, as described under Material and Methods. Subsequently, calcein-AM labelled LS 180 cells were seeded to wells in the presence of different concentrations of *P. nigra* HS or heparin for 1 h at 4 °C. After removing unbound cells, the fluorescence emitted by adherent cells was quantified using a microplate reader. The IC_50_ for the biding of *P. nigra* HS and heparin to P-selectin were calculated as 2.14 µg·mL^−1^ or 42.8 nM and 8.62 µg·mL^−1^ or 507 nM, respectively.

The anticoagulant activity of the *P. nigra* HS was evaluated by the aPTT assay using human plasma in the presence of increasing concentrations of the ascidian glycan (Figure 6A). Using a parallel standard curve based on the aPTT activity of a porcine heparin with 180 IU·mg^−1^, the anticoagulant activity of the tunicate HS was estimated as ~3 IU·mg^−1^ (Figure 6A), 60-fold lower than that of porcine heparin. Similar results were obtained based on anti-factor IIa assay (not shown). This low anticoagulant activity is probably due to the lack of structural components required for the interaction of heparin/HS with the serpin and proteases of the coagulation system. In particular, the overall low sulfation of the *P. nigra* HS will prevent its interaction with the proteins of the coagulation system. Furthermore, the absence of 3-sulfated α-GlcN (Table 1), which is an important component of the antithrombin-binding pentasaccharide, contributes to the reduced anticoagulant activity of the ascidian HS.

### 2.4. Heparan Sulfate from P. nigra Inhibits the Binding of Tumor Cells to Immobilized P-Selectin

P-selectin is a member of a family of calcium-dependent type I transmembrane glycoproteins involved in cell–cell interactions in different pathological conditions, including cancer metastasis and inflammation [18,19]. Once in the bloodstream, cancer cells are covered by platelets, in a P-selectin-mediated process, forming a natural barrier against the immune system [18]. On the other hand, leukocytes recruitment during inflammation is mediated by p-and l-selectins. These adhesion molecules are involved in the first steps of cellular recruitment by reducing the rolling velocity of leukocytes, contributing to their adhesion and arrest at sites of inflammation. In fact, P-selectin null mice exhibit reduced platelet aggregation, delayed leukocyte recruitment, and attenuated metastasis, suggesting that p-selectin might be a common therapeutic target to treat cancer-related inflammation [18,20,21].

Based on the fact that heparin is a potent inhibitor of P-selectin activity [22], we evaluated whether the *P. nigra* HS could inhibit P-selectin binding to tumor cells. For this purpose, we analyzed the ability of this compound to impair adhesion of LS 180 cells to immobilized P-selectin. It is known from previous work that this colon cancer cell line expresses a high content of selectin ligands [23]. The ascidian HS decreased tumor cell binding to P-selectin in a dose-dependent manner, with an IC_50_ of 2.14 μg.mL^−1^ or 42.8 nM, which is 11-fold more efficient than porcine heparin (IC_50_ of 8.62 μg.mL^−1^ or 507 nM) (Figure 6B). It is worth mentioning that because porcine heparin has a lower molecular weight (17 kDa) than the ascidian HS (∼50 kDa, see Figure 2B), the molar concentration of the latter to achieve the biological effect is much less than that of the former, making the invertebrate glycan an even more efficient therapeutic compound.

P-selectin-mediated interactions occur during dissemination of metastatic carcinoma cells. Along this process, in the bloodstream, tumor cells are covered with platelets in a P-selectin-dependent manner. This interaction confers tumor cells with physical shielding mediated by platelets, avoiding natural killer cell-mediated tumor cell lysis, for instance. Based on its potent anti-P-selectin activity, the *P. nigra* HS likely attenuates the seeding of tumor cells to metastatic sites. This is currently under investigation in our laboratory.

Here we described a unique type of HS formed by a variety of α-GlcN units with distinct degrees of *N*-sulfation and *N*-acetylation. The hexuronic acid units occur both as α-Ido and β-GlcA, with variable sulfation at the position 2. A peculiar structural aspect of the tunicate HS is the high content of 2-sulfated β-GlcA units, which accounts for one-third of the total hexuronic acid units. Another distinct aspect of this glycan is the occurrence of a high content of *N*-acetylated α-GlcN units bearing a sulfate group at position 6. This unique ascidian HS is a potent inhibitor of the binding of human colon adenocarcinoma cells to P-selectin, being much more potent than mammalian heparin, but almost ineffective as an anticoagulant, offering an interesting and potential alternative to mammalian heparin as a therapeutic compound in P-selectin-mediated events such as carcinoma metastasis.

Another interesting activity of heparin, which deserves future investigation, is its interaction with proangiogenic factors such as basic fibroblast growth factor (bFGF) and certain isoforms of vascular endothelial growth factor (VEGF). Administration of non-anticoagulant heparin analogs into the tumor site could modulate the bioavailability of proangiogenic factors to their high-affinity receptors, reducing tumor angiogenesis [24,25,26]. However, due to the complexity involved in an antiangiogenic therapy and due to the negative effect on wound healing and vascular remodeling, the risks and benefits of such therapy should be carefully assessed.

Ascidians, that are the primary source of this potential therapeutic compound, abound in different parts of the world. In general, the ascidian glycans occur in high concentration in the tissue (about 0.5% of the dry weight, comparing to 0.022% from pig intestinal mucosa) and can be easily isolated by procedures similar to those already employed in the preparation of pharmaceutical heparin [27]. The use of this marine organism as a source of new non-anticoagulant heparin analogues to provide a significant therapeutic effect depends greatly on the possibility of cultivating them at a large scale to provide the necessary amount of raw material for the production of the biopharmaceutical, just as the process of heparin production from cattle breeding. In fact, the ascidian *Ciona intestinalis*, which as *P. nigra* is a phlebobranchia ascidian, has been cultivated in large scale in Sweden and Norway. The possibility of using cultivated ascidians as a source of potent biopharmaceutical to treat diseases adds huge value to this type of activity, opening a diversity of possibilities in social and environmental areas associated to the rational use of the oceans.

## 3. Material and Methods

### 3.1. Extraction of Sulfated Glycans from the Ascidian Phallusia Nigra

Ascidians *P. nigra* were collected in Angra dos Reis, Rio de Janeiro, Brazil (SISBIO License No 33114-1) and immersed immediately in ethanol. The viscera of the ascidians were removed from the tunic, cut in small pieces, immersed in acetone, and kept for 24 h at 4 °C. Dried material (10 g) was suspended in 500 mL of 0.1 M sodium acetate (pH 5.5), containing 5 g of papain, 5 mM EDTA, and 5 mM cysteine and incubated at 60 °C overnight. The incubation mixture was then centrifuged (2000× *g* for 10 min at room temperature), the supernatant was separated, and this procedure was repeated four more times with the residual solid material, as described above [6]. Supernatants from the five extractions were individually mixed with a solution of cetylpyridinum chloride (CPC) in water (final concentration 0.5%, *w/v*) overnight at room temperature. The precipitate formed was washed with 0.05% (*w/v*) CPC and suspended with 2 M (*w/v*) NaCl. The sample was then mixed with two volumes of 95% (*v/v*) ethanol and kept overnight at 4 °C. The precipitate obtained afterwards (containing the total glycans) was washed with absolute ethanol and dried at 60 °C. Glycans obtained in each one of the five extractions were dissolved in distilled water and analyzed by agarose gel electrophoresis separately.

### 3.2. Fractionation of Sulfated Glycans from P. nigra

#### 3.2.1. Differential Precipitation with Ethanol

Sulfated glycans obtained in different extractions were pooled, lyophilized, suspended in a solution of 2% (*w/v*) NaCl, and fractionated by differential precipitation with ethanol. The solution containing the glycans was mixed with absolute ethanol to achieve a final concentration of 23% (*v/v*) and kept at 4 °C overnight. After centrifugation, the precipitate was collected and the supernatant was mixed with absolute ethanol to achieve a final concentration of 28% (*v/v*). This process was repeated, increasing ethanol concentration to 37.5% (*v/v*) and 50% (*v/v*) at the end. Finally, all precipitates obtained at different concentrations of ethanol were dried and suspended in distilled water for further analysis.

#### 3.2.2. Ion-Exchange Chromatography

The glycans from the second and third extractions were pooled (~20 mg) and applied to a DEAE-cellulose column (GE Healthcare Life Sciences, United Kingdom), linked to a FPLC Äkta Prime system (GE Healthcare Life Sciences, United Kingdom). The column was equilibrated with 0.5 M sodium acetate (pH 6.0) and washed with 50 mL of the same buffer. The column was developed by an increasing concentration of NaCl (0.5→1.0 M) in the same buffer. The flow rate of the column was 8.0 mL h^−1^, and fractions of 1.5 mL were collected and analyzed by metachromasia using 1,9-dimethylmethylene blue [28]. The NaCl concentration was estimated by conductivity. Fractions under the peaks were pooled, dialyzed against distilled water, lyophilized, and analyzed by agarose gel electrophoresis.

### 3.3. Electrophoresis

#### 3.3.1. Agarose Gel

Crude or purified glycans from *P. nigra*, before or after incubation with specific heparin lyases were analyzed by agarose gel electrophoresis, as described previously [6]. Briefly, glycans and a mixture of standard glycosaminoglycans containing CS, DS, and mammalian unfractionated heparin (UFH) (1.5 µg of each) were applied to a 0.5% (*w/v*) agarose gel in 0.05 M 1,3-diaminopropane/acetate (pH 9.0) and ran for 1 h at 110 mV. After electrophoresis, glycans were fixed with aqueous 0.1% (*w/v*) cetylmethylammonium bromide solution and stained with 0.1% (*w/v*) toluidine blue in acetic acid/ethanol/water (0.1:5:5, *v/v/v*).

#### 3.3.2. Polyacrylamide Gel

The molecular weight of purified HS isolated from *P. nigra* was estimated by polyacrylamide gel electrophoresis. The sample (10 µg) was applied to a 1-mm-thick 6% (*w/v*) polyacrylamide slab gel, and after electrophoresis at 100 V for 1 h in 0.06 M sodium barbital (pH 8.6), the gel was stained with toluidine blue. The molecular mass markers used in this assay were: dextran sulfate 500 (average molecular weight, ~500 kDa), CS from shark cartilage (~54 kDa), CS from whale cartilage (~36 kDa), dextran sulfate 8 (~8 kDa), low molecular weight heparin (~8 kDa), and unfractionated heparin (~17 kDa). All markers were purchased from Sigma/Aldrich, MO, USA.

### 3.4. Incubation with Heparin Lyases

Purified HS from *P. nigra* (approximately 20 µg) was incubated with 0.1 unit of heparinase I or III in 100 mM sodium acetate (pH 7.0) containing 10 mM calcium acetate for 17 h at 37 °C. At the end of the incubation period, mixtures were analyzed by agarose gel electrophoresis, as described before [29,30].

### 3.5. NMR

^1^H and ^13^C 1D and 2D spectra of the *P. nigra* HS were recorded at 310 K using a DRX 900 MHz spectrometer (Bruker) with a triple-resonance probe, as described in Reference [31]. For 2D phase-sensitive ^1^H-^1^H TOCSY (phase TOCSY) and ^1^H-^1^H NOESY experiments, spectra were recorded using states-time proportion phase incrementation (States-TPPI) for quadrature detection in the indirect dimension. Phase TOCSY spectra were run with 2048 × 1024 points with a spin lock field of 4 kHz and a mixing time of 80 ms. NOESY spectra were recorded with 2048 × 1024 points and a mixing time of 250 ms. Multiplicity-Edited and non-Edited ^1^H/^13^C HSQC (Ed-HSQC) spectra were recorded as described in Reference [31].

### 3.6. In Vitro Anticoagulant Activity

A mixture of 100 μL of human plasma and various concentrations of *P. nigra* HS or heparin standard was incubated with aPTT reagent (kaolin bovine phospholipid reagent from Biolab-Merieux AS, Rio de Janeiro, Brazil). After 2 min of incubation at 37 °C, 25mM CaCl_2_ (100 μL) was added to the mixtures and the clotting time recorded in a coagulometer KC4A (Heinrich Amelung GmbH). Results were expressed as the ratio of clotting time in the presence (T1) and absence (T0) of different glycosaminoglycan concentrations. The anticoagulant activity was estimated as IU·mg^−1^ using a parallel standard curve based on the 6th International Heparin Standard, obtained from the NIBSC (Potters Bar, UK) [11].

### 3.7. In Vitro Binding of LS 180 Cells to Immobilized P-Selectin

The ability of *P. nigra* HS to inhibit adhesion of tumor cells onto immobilized P-selectin chimeras was assessed as described before [32]. For this, LS 180 cells (human colorectal adenocarcinoma cell line purchased from ATCC, Manassas, VA, USA) were grown in minimum essential medium-α (Invitrogen, CA, USA) supplemented with 10% (*v/v*) fetal bovine serum (FBS) (Invitrogen, CA, USA). 96-well microplates were coated with protein A and then blocked with 1% (*w/v*) BSA in HBSS buffer (blocking buffer). Subsequently, P-selectin chimera (400 ng/well) was added to the wells, incubated for 3 h at room temperature, and then washed with blocking buffer (3 times). Then, calcein-AM labelled LS 180 cells (5 × 10^4^ cells/well) were seeded to wells in the presence of different concentrations of *P. nigra* HS or heparin for 1 h at 4 °C. After removing unbound cells, the fluorescence emitted by adherent cells was quantified using a microplate reader (Tecan; 485 nm for excitation and 520 nm for emission). IC_50_ of *P. nigra* HS was calculated according to three independent experiments.

## Figures and Tables

**Table 1:** Proportions (mean ±SD) and chemical shifts of the constitutive units of the *P. nigra* heparan sulfate determined via solution NMR.

Signal ^a^	Structure ^b^	Proportion ^c^	^1^H/^13^C Chemical Shift (ppm)					
			1	2	3	4	5	6
**(A) α-GlcN Units**								
Na	**αGlcN(NS,6S)**→[βGlcA(2S)]	5.53 ± 1.40	5.57/97.7	3.28/57.9	3.70/69.7	*3.93/72.7*	4.05/70.8	**4.20/69.4**
Nb	**αGlcN(NS)**→[βGlcA(2S)]	15.30 ± 1.27	5.56/97.7	3.26/57.9	3.68/67.8	*3.97/72.9*	4.01/70.9	3.67/60.1
Nc	**αGlcN(NS,6S)**→[αIdoA(2S)]	13.30 ± 0.13	5.42/96.8	3.28/57.9	3.66/69.8	*3.89/75.2*	3.97/69.2	**4.25/66.2**
Nd	**αGlcNAc**→[βGlcA]	14.39 ± 0.29	5.38/96.8	3.88/55.7	3.71/69.7	*3.77/68.2*	3.85/69.1	3.78/61.0
Ne	**αGlcN(NS)**→[αIdoA(2S)]	12.48 ± 2.01	5.31/97.0	3.28/57.9	3.73/71.1	*3.75/77.3*	3.89/75.2	3.87/59.7
Nf	**αGlcNAc(6S)**→[βGlcA]	39.00 ± 2.92	5.14/101.1	4.02/51.2	3.70/69.8	*3.77/68.2*	3.92/70.7	**4.20/67.5**

**(B) α-IdoA Units**								
Ia	**αIdoA(2S)**→[αGlcN(NS)]	3.16 ± 0.24	5.23/99.3	**4.33/74.8**	4.23/69.3	*4.05/75.7*	4.83/71.1	-
Ib	**αIdoA(2S)**→[αGlcN(NS,6S)]	5.93 ± 0.33	5.24/99.3	**4.34/76.0**	4.23/69.3	*4.10/76.0*	4.80/72.7	-
Ic	**αIdoA(2S)**→[αGlcN(NS)]	10.46 ± 2.91	5.16/101.1	**4.33/74.8**	4.16/73.5	*4.08/77.0*	4.54/67.1	-
Id	**αIdoA**→[αGlcNAc(6S)]	13.42 ± 2.06	5.04/101.3	3.78/75.8	3.37/73.4	*4.07/75.9*	4.81/69.5	-
Ie	**αIdoA**→[αGlcN(NS)]	11.18 ± 1.95	4.98/100.4	3.81/76.6	3.41/73.5	*4.01/77.0*	4.84/68.4	-
	**ΔIdoA**	**44.15**						
**(C) β-GlcA Units**								
Ua	**βGlcA(2S)**→[αGlcN(NS)]	17.35 ± 0.09	4.60/102.0	**4.00/77.3**	3.73/75.1	*3.84/75.8*	3.87/74.9	-
Ub	**βGlcA(2S)**→[αGlcN(NS,6S)]	16.37 ± 0.86	4.60/103.6	**4.01/78.3**	3.74/77.4	*3.82/75.8*	3.88/75.8	-
Uc	**βGlcA**→[αGlcNAc]	11.81 ± 2.92	4.52/102.4	3.40/72.9	3.70/76.4	*3.86/77.1*	3.89/75.2	-
Ud	**βGlcA**→[αGlcNAc(6S)]	10.33 ± 0.16	4.52/103.3	3.47/72.8	3.70/77.3	*3.85/74.9*	3.90/75.3	-
	**ΔGlcA**	**55.85**						

^a^ See panels B and C, Figure 2. ^b^ The reported structure is in bold and the subsequent unit is shown between brackets. ^c^ Results are expressed as percentage of the total GlcN or hexuronic acid units (IdoA + GlcA) calculated via integral (absolute values) after deconvolution of the ^1^H/^13^C anomeric signals in the quantitative HSQC spectra (see Material and Methods section). The proportion of the superimposed GlcN and IdoA residues was calculated by integration of their H6/C6 and H2/C2, respectively, subtracted from their correspondent anomeric signal. Chemical shifts in bold represent the presence of *O*-sulfation sites and those in italic represent the presence of glycosidic bonds.
